# Conjunctivitis can be a possible clinical presentation of monkeypox

**DOI:** 10.5935/0004-2749.2022-0194

**Published:** 2025-08-22

**Authors:** Rujittika Mungmunpuntipantip, Viroj Wiwanitkit

**Affiliations:** 1 Private Academic Consultant, Bangkok, Thailand; 2 DY Patil University, Pune, India

Dear Editor,

Smallpox and chickenpox are the two most common human pox infections. In addition to these diseases, novel zoonotic pox diseases have been a major concern in infectious medicine^(1)^. Monkeypox has spread throughout Europe, thereby causing a significant public health risk^(2)^. It is a rare pox infection that has resurfaced and is most likely caused by zoonosis^(1)^. Human-to-human transmission is currently investigated. The medical community is concerned as the number of cases reported is increasing in different countries.

Based on our experience with coronavirus disease 2019, we must respond promptly and investigate the problem appropriately in case of an epidemic. Moreover, we should act immediately to comprehensively investigate the issue and take the required management steps^(2)^. Conjunctivitis is a common clinical symptom. However, it can be observed in any novel infectious diseases and can be the only clinical presentation (similar to coronavirus disease 2019)^(3)^. In 2022, the number of new monkeypox cases in large clusters in several countries outside of Africa is rapidly increasing, thereby causing concerns on a statewide outbreak.

Monkeypox is an illness that causes fever and skin rash. The initial diagnosis is commonly made based on the presence of rash. To the best of our knowledge, no previous research has investigated the symptoms of monkeypox. Conjunctivitis was the presenting symptom in 3 of 34 patients who were evaluated. These individuals did not develop fever, and some had a rash^(4)^. Atypical characteristics and the absence of a cutaneous lesion and fever are the clinical features of monkeypox that can possibly develop but are under-reported^(1)^. Conjunctivitis can be the initial symptom. Hence, emergency preparedness is important in the current clinical practice. Further, medical professionals should be alert for suspected monkeypox cases since an epidemic in a new setting can occur, and all patients with conjunctivitis must be evaluated comprehensively.
